# Long non-coding RNAs in brain tumors

**DOI:** 10.1093/narcan/zcaa041

**Published:** 2021-01-15

**Authors:** Keisuke Katsushima, George Jallo, Charles G Eberhart, Ranjan J Perera

**Affiliations:** Department of Oncology, Johns Hopkins University School of Medicine, 1650 Orleans St., Baltimore, MD 21231, USA; Johns Hopkins All Children's Hospital, 600 5th St. South, St Petersburg, FL 33701, USA; Johns Hopkins All Children's Hospital, 600 5th St. South, St Petersburg, FL 33701, USA; Department of Neurosurgery, Johns Hopkins University School of Medicine, 600 N Wolfe St., Baltimore, MD 21287, USA; Department of Oncology, Johns Hopkins University School of Medicine, 1650 Orleans St., Baltimore, MD 21231, USA; Department of Pathology, Johns Hopkins University School of Medicine, 720 Rutland Ave Ross Bldg 558, Baltimore, MD 21205, USA; Department of Oncology, Johns Hopkins University School of Medicine, 1650 Orleans St., Baltimore, MD 21231, USA; Johns Hopkins All Children's Hospital, 600 5th St. South, St Petersburg, FL 33701, USA

## Abstract

Long non-coding RNAs (lncRNAs) have been found to be central players in the epigenetic, transcriptional and post-transcriptional regulation of gene expression. There is an accumulation of evidence on newly discovered lncRNAs, their molecular interactions and their roles in the development and progression of human brain tumors. LncRNAs can have either tumor suppressive or oncogenic functions in different brain cancers, making them attractive therapeutic targets and biomarkers for personalized therapy and precision diagnostics. Here, we summarize the current state of knowledge of the lncRNAs that have been implicated in brain cancer pathogenesis, particularly in gliomas and medulloblastomas. We discuss their epigenetic regulation as well as the prospects of using lncRNAs as diagnostic biomarkers and therapeutic targets in patients with brain tumors.

## INTRODUCTION

### Central nervous system tumors

Gliomas represent about a third of all central nervous system (CNS) tumors and include astrocytomas, oligodendrogliomas, ependymomas and glioblastomas. Despite progress in surgery, radiotherapy and chemotherapy, many glioma patients have a poor prognosis (1); for example, the median survival for patients with high-grade glioma is only 15 months (2). A better understanding of the characteristics and pathobiology of gliomas is necessary to improve tumor classification and patient stratification for more targeted treatment. There have been substantial efforts to characterize the gene expression, mutational and epigenetic landscapes of gliomas, not least for the purposes of biomarker identification (3). As a direct result of these efforts, a new and improved glioma classification system that includes molecular biomarkers was approved in 2016 (4). Diffuse astrocytic and oligodendroglial tumors (WHO grade II and III) are now separated according to *IDH* mutations and 1p/19q-codeletion status, while grade IV gliomas are now divided into *IDH* wild-type, *IDH*-mutant, glioblastoma not otherwise specified and diffuse midline glioma, *H3 K27M*-mutant. In adults, about 32% of brain tumors are malignant, of which about half are glioblastomas, about one-fifth are diffuse astrocytomas and about one in twenty are oligodendrogliomas and ependymal tumors (5).

In children, medulloblastomas (MBs) are the most common brain tumors (6–8), representing ∼20% of pediatric brain tumors but only 1% of adult cases (6,9). MBs are poorly differentiated ‘embryonal’ tumors arising in in the cerebellum and are highly malignant; they commonly metastasize to other parts of the brain and to the spinal cord and, occasionally, to extraneural sites (10,11). Transcriptional programs in MBs have been shown to mimic developmental cerebellar lineages, highlighting their embryonic origin (12). The clinical management of MBs depends on several factors including molecular and histopathological tumor subtype, stage and extent of resection, location and overall patient health. Treatment strategies tend to be aggressive, consisting of a mixture of surgical resection, radiotherapy, chemotherapy and stem cell/bone marrow transplantation. Despite advances in their diagnosis and treatment, MB remains deadly in 35–40% of cases and those patients that do survive often suffer long-term side-effects including organ dysfunction, neurocognitive impairment, endocrine disabilities and secondary tumors (13–16). In the major 2016 WHO re-classification of CNS tumors, MBs were subdivided into following major molecular subgroups (8): wingless-type (WNT)-activated MBs (10%; children and adults, associated with very good prognosis); sonic hedgehog (SHH)-activated MBs (30%; intermediate prognosis, infants and adults; further characterized as *TP53* mutant or *TP53* wild-type); and two provisional non-WNT/SHH subgroups: group 3 MBs (25%; poor prognosis, infants and children) and group 4 MBs (35%; intermediate prognosis, children and adults) (reviewed in (17,18)). Despite this progress, the underlying subtype-specific pathobiology and molecular processes in MB are not fully defined, hampering the exploitation of subgrouping to personalize therapy for individual patients.

### Long non-coding RNAs

Given rapid developments in genome and transcriptome sequencing technologies and the implementation of genomics consortia such as ENCODE and FANTOM, the classical, mRNA-centric view of the transcriptomic landscape has undergone fundamental changes (19–21). The genome serves as a template not only for coding RNAs but for also a far greater number of non-coding RNAs (ncRNAs). Of the non-coding RNAs, long non-coding RNAs (lncRNAs), which are >200 nucleotides in length, have been widely investigated and identified as key regulators of various biological processes, including cellular proliferation, differentiation, apoptosis, migration and invasion (22–25). Recent studies have shown that lncRNAs play key roles in a wide range of cellular processes by regulating gene expression at the epigenetic, transcriptional and post-transcriptional levels (26–28). LncRNAs may be classified into several archetypes based on their mode of action and functions:

- *Molecular signals*. LncRNAs can function as molecular signals to induce transcriptional activity. LncRNAs display tissue-specific expression and respond to varied cellular stimuli, highlighting their tight transcriptional control. They can act as molecular signals through exquisite orchestration of their transcription and subcellular location, allowing the integration of responses to different stimuli (29).
- *Molecular decoys*. LncRNAs can act as molecular ‘sponges’ of RNA-binding proteins such as chromatin remodelers, transcription factors and other regulatory factors. This mechanism plays a central role in both positive and negative transcriptional regulation by lncRNAs (30).
- *Molecular guides*. LncRNAs direct the localization of ribonucleoprotein (RNP) complexes to specific targets. LncRNAs act as guides, directly binding to proteins and thus altering the location of RNPs to target regions, leading to changes in gene expression. The regulatory components triggered by the lncRNAs include both repressive and activating complexes as well as transcription factors (31–33).
- *Scaffolds*. LncRNAs can serve as central platforms for protein complex assembly by binding distinct effector molecules. LncRNAs possess distinct protein-binding domains for binding multiple effector components (which may be transcriptional activators or repressors) in both time and space to regulate gene transcription or repression (33,34).
- *Competing endogenous RNAs (ceRNAs)*. Some evidence shows that lncRNAs may be potent natural microRNA (miRNA) sponges. LncRNAs containing miRNA binding sites de-repress miRNA target genes through complementary binding to miRNAs (35).
- *Enhancers*. Enhancers are a signature feature of the regulation of gene expression during development and play a key role in specifying cell identity. There are up to 400 000 enhancers in the human genome, many clustered as so-called ‘super-enhancers’(36). Enhancers transcribe to produce noncoding RNAs specifically in the cells in which they are active and appear to be integral to enhancer action by looping of regulatory elements to affect target gene transcription and the formation of topologically associated domains in the process (36,37).

**Figure 1.**
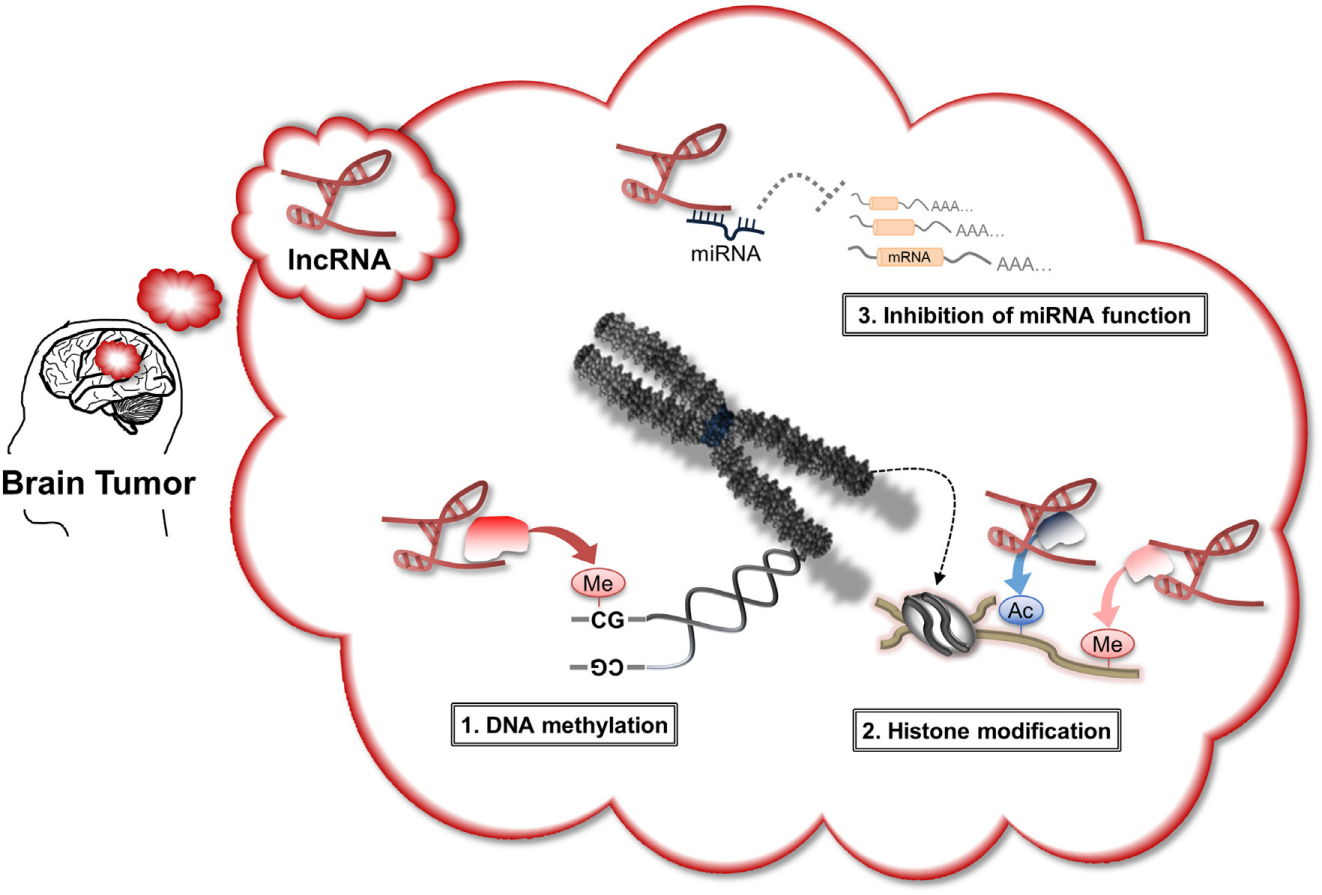
The multiple dimensions of lncRNA function in brain tumors. (**1**) LncRNAs interact with DNA methyltransferases (DNMTs), resulting in the recruitment or inhibition of DNMTs at chromatin loci. Alterations in DNA methylation levels generally affect local transcription. (**2**) LncRNAs acting as scaffolds bind to chromatin and epigenetic modifiers to guide the epigenetic modifier to gene promotors. (**3**) LncRNAs acting as miRNA sponges attenuates miRNA effects on downstream mRNA expression.

Dysregulated lncRNA expression could potentially alter basic cell biology and contribute to tumorigenesis of all the tumors listed above (38–40). LncRNAs are widely expressed in the brain and during neurological development, mostly in highly specific patterns and cell types (41,42) and play important roles in brain tumor pathogenesis via multiple gene expression control mechanisms (Figure 1). In this review, we explore the expression profiles and functions of lncRNAs in brain tumors, especially gliomas and MBs, and discuss their potential use as diagnostic and prognostic biomarkers and therapeutic targets.

## ONCOGENIC AND TUMOR SUPPRESSOR LNCRNAS IN BRAIN TUMORS

### Oncogenic lncRNAs in gliomas

*LncRNA-ATB* is a lncRNA induced by TGF-β (ATB) that is widely expressed in different types of cancer (43). It functions in part through upregulation the zinc finger and homeodomain-containing transcription factors ZEB1 and ZEB2 by competitively binding the *miR-200* family and inducing endothelial-mesenchymal transition and invasion. It also promotes organ colonization of disseminated tumor cells by binding IL-11 mRNA, autocrine induction of IL-11 and triggering STAT3 signaling (44). The expression of *lncRNA-ATB* positively correlates with glioma grade and is negatively associated with survival in glioma patients. *LncRNA-ATB* is targeted by *miR-200a*, which affects *TGF-β2* (transforming growth factor-beta 2) expression. In turn, TGF-β2 activates the MAPK (mitogen-activated protein kinase) signaling pathway (45). Downregulation of *lncRNA-ATB* suppresses tumor growth *in vivo* (46).

*H19* lncRNA was first described as a parentally imprinted lncRNA expressed from the *IGF2* (insulin-like growth factor 2) locus (47) that plays a role in fetal growth and tumor etiology (48), in part by controlling DNA methylation (49). Expression modulates glioma growth by targeting iASPP (inhibitor of apoptosis-stimulating protein of p5) via *miR-140* (44). The pro-proliferative effect of *H19* is mediated by *miR-675*, which is encoded in *H19* exon 1 (50) and regulates CDK6 (cyclin-dependent kinase 6) expression (51). *H19* also downregulates *miR-152* (52), leading to tumor proliferation and invasion *in vitro* and *in vivo*. *H19* has also been shown to function as a ceRNA to regulate epithelial-to-mesenchymal transition (EMT) by sponging *miR-130a-3p* (53).

The lncRNA *CCAT1* is involved in cellular proliferation and is a *miR-410* target (54). *miR-410* expression was found to be suppressed in glioma tissue, leading to MET/Akt pathway activation (55). *CCAT1* enhanced *FGFR3* (fibroblast growth factor receptor 3) and *PDGFRA* (platelet-derived growth factor receptor A) expression by targeting *miR-181b*; both growth factors activate MAPK signaling (55). *CCAT1* also promoted tumor growth *in vivo* (56).

*CRNDE* lncRNA was found to be highly upregulated in glioma tissue and it promoted tumor proliferation, migration and invasion (57). CRNDE induced gliomagenesis *in vivo*. CRNDE is a *miR-384* target, and both regulate PIWIL4 (piwi-like RNA-mediated gene silencing 4) protein, which promotes progression by inducing STAT3 (signal transducer and activator of transcription 3) phosphorylation (58). Upregulated expression of *CRNDE* induces EGFR (epidermal growth factor receptor) activation, resulting in apoptosis inhibition through an increased Bcl2 (B-cell CLL/lymphoma 2)/Bax (BCL2-associated X) ratio and an association with poor survival in glioma patients (59).

Several studies have shown that *HOTAIR* lncRNA is an *miR-326* (60) and *miR-148b-3p* (61) target. Suppressing *HOTAIR* expression, together with mimics of *miR-326*, strongly inhibited glioma cell proliferation, migration and invasion. *miR-326* targeted FGF and activated the phosphoinositide 3-kinase (PI3K)/protein kinase B (AKT) signaling pathway (62). The involvement of *HOTAIR* and *miR-326* in gliomagenesis was also demonstrated *in vivo* (60,63). *HOTAIR* inhibition also induced apoptosis, and the BET family protein BRD4 (bromodomain-containing 4)-regulated *HOTAIR* expression (64).

Decreased *HOXA11-AS* lncRNA expression induced cell cycle arrest and initiated apoptosis in glioma cell lines. *HOXA11-AS* is targeted by *miR-140–5p* (65), which regulates cell proliferation and invasion via the VEGFA (vascular endothelial growth factor A)/MMP-2 (matrix metalloproteinase 2) signaling pathway (66). Recently, Xu *et al.* showed that *HOXA11-AS* reduced *miR-214–3p* expression, which directly targeted EZH2 (enhancer of zeste homolog 2) (67).

*UCA1* lncRNA is involved in proliferation and migration, and its expression is positively associated with overall glioma patient survival. *UCA1* activates expression of inhibitor of apoptosis stimulating protein of p53 (iASPP) by inhibiting *miR-182* expression (68). In normal brain tissue, *miR-182* regulates apoptosis, proliferation and migration by inhibiting iASPP expression (69). Elevated *UCA1* downregulates *miR-122* (70), which in turn associate with proliferation, invasion and migration via Wnt/β-catenin signaling (71–73). In addition, inhibition of *UCA1* promotes *CCND1* (cyclin D1) expression (74).

*NEAT1* lncRNA is a widely expressed lncRNA that is essential for the formation of paraspeckles, enigmatic mammalian-specific subnuclear organelles that are important for placental biology and may act as reservoirs of edited transcripts and/or transcripts that are important for stress responses (75). *NEAT1* has associated with epilepsy (76), and paraspeckle components are implicated in motor neuron disease (75). *NEAT1* overexpression in glioma tissue positively correlates with glioma grade (77), and reduced *NEAT1* expression is associated with longer survival in glioma patients (78). *NEAT1* activates proliferation, migration and invasion and deregulates apoptosis, possibly by interacting with miR-449b-5p, which activated the *MET* oncogene (77). In addition, *NEAT1* is a direct target of the miRNA *let-7e* and activates cellular proliferation via PI3K/AKT/mTOR (mammalian target of rapamycin) and mitogen-activated protein kinase (MAPK)/extracellular-signal-regulated kinase (ERK) signaling (79). *NEAT1* also activates MET by silencing *miR-449b-5p* (77). Recently, Yang *et al.* discovered that *NEAT1* increased CDK6 (cyclin-dependent kinase 6) expression via *miR-107* in glioma cell lines (80). On the other hand, *PVT1* lncRNA expression is increased in glioma tissue, is positively associated with poor outcomes and alters *EZH2* expression. *PVT1* also regulates cell cycle and apoptosis *in vitro* and *in vivo* (81,82). *PVT1* regulates the expression of *MEF2C* (myocyte enhancer factor 2C) and *JAG1* (Jagged1) by binding to *miR-190a-5p* and *miR-488–3p* (83).

*TUG1*, a lncRNA first identified as being involved in retinal development, is widely expressed in the brain. It is also expressed in T cells (84) and is required for male fertility (85). *TUG1* also upregulation activates caspases by inhibiting *Bcl-2* expression, suggesting involvement in the regulation of programmed cell death and immune responses (86). Furthermore, *TUG1* maintained glioma stem cells through interactions with polycomb repressive protein 2 (PRC2) components (EZH2 and SUZ12 (SUZ12 polycomb repressive complex 2 subunit)) and transcription factor YY1 (Yin and Yang 1 protein), thereby epigenetically suppressing multiple neuronal differentiation-associated genes (87).

The sex chromosome dosage compensation lncRNA *XIST* is upregulated in glioblastoma cells, and its depletion inhibited glioblastoma angiogenesis via *ZO2* (tight junction protein ZO-2) and *FOXC1* (forkhead box C1) transcriptional inactivation (88). Both ZO-2 and FOXC1 are crucial for maintaining blood–tumor barrier integrity through the upregulation of *miR-137* (88). *XIST* also acts as a molecular sponge for *miR-429* in glioblastoma cells, and the negative regulation of *XIST* contributes to the repression of glioblastoma cell metastatic and angiogenic potential (89). *XIST* depletion also exerts tumor suppressive roles in glioma stem cells by upregulating *miR-152* (90). *ZEB1-AS1* (antisense to ZEB1) lncRNA represses T cell-specific interleukin expression (91)) regulates the expression of genes encoding cyclin D, CDK2 (cyclin-dependent kinase 2), ZEB1 (zinc finger E-box-binding homeobox 1), MMP2, MMP9, N-cadherin and integrin-β1. Therefore, *ZEB1-AS1* regulates EMT and is involved in glioma proliferation, apoptosis and metastasis (92).

*ECONEXIN* lncRNA is upregulated in glioma tissue and promotes cell proliferation by sponging *miR-411–5p* and altering *TOP2A* (DNA topoisomerase II alpha) gene expression (93). Increased expression of *TP73-AS1* lncRNA is associated with poor patient survival, and *TP73-AS1* downregulation inhibits proliferation and invasion as well as *HMGB1* (high mobility group box 1) expression by targeting *miR-142* (94,95). Decreased expression of miR-142 correlates with increased levels of *RAC1* (Rac family small GTPase 1), which activates multiple oncogenic pathways (96).

*FOXD3-AS1* lncRNA has been shown to be involved in cell proliferation, migration and invasion, associated with poor prognosis and correlated with glioma grade. *FOXD3-AS1* overexpression reduces transcription factor FOXD3 (forkhead box D3) levels to participate in differentiation, proliferation, migration and apoptosis (97). *ZFAS1* lncRNA activates cell proliferation, invasion and migration by activating EMT and Notch signaling pathways (98). *Linc-OIP5* lncRNA is upregulated in glioma tissue and positively correlates with glioma grade. *Linc-OIP5* induces proliferation and migration through NOTCH1 (Notch homolog 1, translocation-associated), YAP (yes-associated protein 1), JAG1 and HES1 (hairy and enhancer of split-1) and downregulating its expression reduced tumor growth *in vivo* (99).

### Tumor suppressive and context-dependent lncRNAs in gliomas

The majority of available evidence shows that the expression of the lncRNA *HOTTIP* is mainly downregulated in gliomas. However, its overexpression inhibited proliferation and induced apoptosis *in vitro*. Also, there was a negative correlation between *HOTTIP* and *BRE* (BRCA1-A complex subunit) (100). *BRE* plays an essential role in preventing replicative and DNA damage-induced premature senescence (101). In addition, reduced *BRE* expression downregulated *CCNA* (cyclin A) and *CDK2* expression and increased p53 expression (100). Interestingly, another study showed that *HOTTIP* was overexpressed under hypoxic conditions, and *HOTTIP* expression in metastatic glioma samples was higher than in glioma samples without metastasis. Zhang *et al.* demonstrated that *HOTTIP* contributed to EMT and metastasis in glioma by targeting *miR-101* and elevating *ZEB1* transcription, itself a target of *miR-101* (102).

*MALAT1* lncRNA has been shown to be downregulated in glioma tissue and cell lines, and high *MALAT1* levels are associated with improved survival in glioma patients. *MALAT1* also negatively regulates *miR-155* expression, and FBXW7 (F-box and WD repeat domain containing 7) is a validated target of *miR-155*. *MALAT1* suppresses glioma cell viability by downregulating *miR-155* and promoting *FBXW7* expression (103). Han *et al.* also measured *MALAT1* expression in glioma specimens and cell lines and found that *MALAT1* expression is higher in non-cancerous brain tissues, albeit with no differences between grades. Overexpression of *MALAT1* is associated with MAPK pathway suppression (104).

In apparent contradiction, *MALAT1* expression has been shown by others to be highly expressed in glioma tissue and cell lines (105). *MALAT1* knockdown suppresses glioma cell proliferation and induces apoptosis, together with downregulated *CCND1* and *MYC* expression (105). Fu *et al.* also detected an increase in *MALAT1* expression in gliomas, concluding that *MALAT1* can induce proliferation and autophagy and target *miR-101*, which regulates not only ZEB1, but also STMN1 (stathmin 1), RAB5A (RAS-associated protein RAB5A) and ATG4D (autophagy-related 4D cysteine peptidase) expression (106). The variable reports of the expression patterns of *MALAT1* in gliomas may be due to the complexity of ncRNA biology and glioma heterogeneity or the vagaries of assessing differential gene expression, which may apply more broadly.

*TUSC7* lncRNA is reported to be downregulated in glioma tissue and negatively associated with overall survival and miR-23b expression (107). In addition, Jiang *et al.* showed that miR-23b regulated the PI3K/Akt signaling pathway (108). Several studies have implicated *MEG3* lncRNA in glioma pathogenesis (109–111). *MEG3* is downregulated in glioma cell lines, and its overexpression increased cell death and inhibited proliferation (110). Other studies have shown that the *MEG3* promoter can be hyper-methylated by DNA methyltransferase 1 (DNMT1) in glioblastoma tissue, thereby completely silencing the gene (112). Additionally, *MEG3* acted as a ceRNA to *miR-19a*, which repressed PTEN (phosphatase and tensin homolog) expression, leading to glioma cell proliferation, migration and invasion (113).

*RAMP2-AS1* lncRNA is downregulated in glioblastoma tissues, and its ectopic overexpression reduces tumor growth in a subcutaneous mouse model (114). This lncRNA interacts with the DHC10/NOTCH3/HES1 signaling pathway and plays a tumor-suppressive role in glioblastoma progression through inhibition of NOTCH3 (114). *CASC2* lncRNA is downregulated in glioma tissues and glioblastoma cell lines (115). *CASC2* overexpression inhibits glioblastoma cell proliferation, migration and invasion via *miR-21*, and *miR-21* overexpression abrogated *CASC2*-induced inhibitory effects (115). Mechanistically, *miR-21* bound to *CASC2* in a sequence-specific manner, leading to the transcriptional repression of *CASC2*. Similarly, *CASC2* overexpression suppresses the Wnt/β-catenin signaling pathway in glioblastoma cells (116). Glioblastoma cells display resistance to temozolomide (TMZ), at least in part, through increased autophagy. Interestingly, TMZ sensitivity was *CASC2*-dependent in glioma stem cells. In one study, *CASC2* overexpression inhibited autophagy and increased glioma stem cell susceptibility to TMZ by the accumulation of lipid peroxides, leading to cell death (117). Autophagy inhibition mediated by *CASC2* upregulation may be due to sponging *miR-193a-5p* and regulating *mTOR* expression (117).

Table 1 summarizes the lncRNAs implicated in glioma and their molecular partners or genomic targets that mediate glioma progression.

**Table 1.** Summary of lncRNAs involved in epigenetic regulation and in glioma biology

lncRNA	Biological roles in glioma	Molecular functions	Target pathway	References
*ADAMTS9‐AS2*	Promotion of cell proliferation, migration and invasion Role in TMZ resistance	Regulated by DNA methylation and DNMT1	mTOR pathway	(178,179)
*CASC2*	Inhibition of cell proliferation, migration and invasion Chemoresistance to TMZ	miRNA sponge (miR-21, miR-181a and miR-193–5p)	Wnt/β-catenin pathway PTEN Pathway	(180–182)
*CCAT1*	Promotion of cell proliferation, migration and invasion	miRNA sponge (miR-181b and miR-410)	MET/Akt pathway MAPK/ERK1 pathway	(54,56)
*CCDC26*	Promotion of cell cycle and proliferation	miRNA sponge (miR-203)	Unknown	(183)
*CRNDE*	Promotes glioma cell growth and migration Regulation of glioma cell stemness	Scaffold with PRC2 and CoREST miRNA sponge (miR-384 and miR-136–5p)	mTOR pathway	(58,184–187)
*ECONEXIN*	Promotion of cell proliferation	miRNA sponge (miR-411–5p)	Unknown	(93)
*FER1L4*	Promotion of cell proliferation and migration	miRNA sponge (miR-372)	Unknown	(188)
*H19*	Promotion of cell proliferation, migration and invasion	miRNA sponge (miR-29a, miR-140 and miR-675)	Unknown	(44,53,189)
*HOTAIR*	Promotes glioma cell growth and stemness Promotion of cell proliferation, migration and invasion	Scaffold with PRC2 and LSD1/CoREST/REST complex regulated by BRD4 miRNA sponge (miR-148b-3p, miR-141, miR-181d-5p and miR-326)	Cell cycle and proliferation pathway	(60,64,144,190–192)
*HOTAIRM1*	Potential roles in glioma‐genesis and development	Scaffold with PRC1 and PRC2	Unknown	(141,193)
*HOTTIP*	Regulation of hypoxia-induced EMT and metastasis	miRNA sponge (miR-101)	ZEB1 pathway	(102)
*HOXA11-AS*	Promotion of cell proliferation, migration and invasion	miRNA sponge (miR-214–3p and miR-140–5p)	VEGFA/MMP-2 signaling pathway WNT/β-Catenin pathway NOTCH pathway	(67,194)
*Linc‐p21*	Inhibition of radiosensitivity promotion of autophagy	Scaffold with SETDB1 and DNMT1	HIF1-mTOR pathway	(49,195,196)
*linc-POU3F3*	Promotion of cell proliferation	Interacts with EZH2	YAP-NOTCH pathway	(197,198)
*Linc‐ROR*	Inhibition of glioma cell growth Inhibition of self‐renewal of glioma cell	Interacts with EZH2	ZEB1 pathway	(199)
*LncRNA-ATB*	Promotion of cell proliferation, migration and invasion	miRNA sponge (miR-200a)	NF‐κB pathway MAPK pathway	(46,91)
*MALAT1*	Inhibition of glioma cell growth Modulates BTB permeability	miRNA sponge (miR-101, miR-140 and miR-155)	MAPK pathway	(103,106,160)
*MEG3*	Inhibition of glioma cell growth Involved in angiogenesis and brain vascularization	Regulated by DNA methylation and DNMT1 miRNA sponge (miR-19a)	p53 pathway PTEN pathway	(112,200)
*NEAT1*	Promotion of cell proliferation, migration and invasion regulation of glioma cell stemness modulates BTB permeability	Scaffolding PRC2 complex (EZH2) miRNA sponge (let-7e, miR-107, miR-132 and miR-449b-5p)	WNT/β-Catenin pathway PI3K/AKT/mTOR pathway MEK/ERK pathway	(77,79,201–204)
*PVT1*	Promotion of cell proliferation, migration and invasion Role in TMZ resistance and radiotherapy response	miRNA sponge (miR-128–3p, miR-190a-5p and miR-488–3p) regulated by EZH2	Cell cycle pathway BMP pathway NOTCH pathway	(82,83,205)
*TP73-AS1*	Promotion of cell proliferation, migration and invasion Role in TMZ resistance	miRNA sponge (miR-142 and miR-124)	Unknown	(206–208)
*TUG1*	Promotion of cell proliferation, migration and invasion regulation of glioma cell stemness enhances VEGF expression and angiogenesis	Scaffold for PRC2 complex, guide for EZH2 miRNA sponge (miR-26a, miR-144 and miR-145)	WNT/β-Catenin pathway EMT pathway Neuronal differentiation pathway	(209–212)
*TUSC7*	Inhibition of cell proliferation, migration and invasion	miRNA sponge (miR-23b)	PI3K/Akt pathway	(107)
*XIST*	Promotion of cell proliferation, invasion, stemness promotion of glioma angiogenesis and BTB Role in TMZ resistance	Scaffold for PRC2 complex miRNA sponge (miR-29c, miR-139, miR-429 and miR-152)	Unknown	(88,90,213,214)
*ZEB1-AS1*	Promotion of cell proliferation, migration and invasion regulation of EMT	miRNA sponge (miR-144, miR-200c and miR-577)	Unknown	(215,216)

### Dysregulated lncRNAs in medulloblastoma

Knowledge of lncRNA function in MB is still fragmentary, and there are only a few articles on the topic. Here we discuss recently reported mechanistic insights into how lncRNAs regulate gene expression and contribute to MB formation.

*Linc-NeD125* lncRNA, also known as *miR100HG*, is significantly overexpressed in group 4 MBs, the largest and least well-characterized medulloblastoma molecular subgroup. Mechanistically, *linc-NeD125* has been reported to recruit the miRNA-induced silencing complex (miRISC) and directly bind to *miR-19a-3p, miR-19b-3p* and *miR-106a-5p* (118). Functionally, *linc-NeD125* appears to act as a miRNA sponge that sequesters these three miRNAs and de-represses their targets *CDK6, MYCN, SNCAIP* (synuclein-alpha-interacting protein), and *KDM6A* (lysine demethylase 6A), which are major driver genes of group 4 MBs. Consistent with the role of *linc-NeD125* as an oncogene, ectopic expression of *linc-NeD125* suppressed medulloblastoma cell proliferation, migration and invasion *in vitro* (118).

*NKX2–2-AS1* lncRNA is involved in Sonic Hedgehog (SHH)-driven MB development. In addition, the SHH pathway transcription factor GLI2 (GLI family zinc finger 2) switches on *FOXD1* (forkhead box D1) expression, which subsequently represses transcription of *NKX2–2-AS1*. Specifically, *NKX2–2-AS1* functions as a miRNA sponge to sequester *miR-103, miR-107* and *miR-548m*, thereby maintaining the expression of their tumor-suppressive targets BTG2 (BTG anti-proliferation factor 2), LATS1 (large tumor suppressor kinase 1) and LATS2. Thus, GLI2/FOXD1-mediated *NKX2–2-AS1* downregulation contributes to the pathogenesis of SHH-subgroup MBs (75).

*PVT1* is a non-coding host gene for four miRNAs (*miR-1204, miR-1205, miR-1206 and miR-1207*) and is amplified together with *MYC* in group 3 MBs. *PVT1* fusion genes are highly recurrent, restricted to group 3 MBs, arise through a chromothripsis-like process and were the first recurrent translocation reported in MB (119). The *PVT1* locus is thought to be genomically fragile, as the majority of *MYC*-amplified group 3 MBs harbor *PVT1* fusions. The identified *PVT1* fusions involve only *PVT1* exon 1 and miR-1204, and only miR-1204 and not the adjacent miR-1205, miR-1206 and miR-1207 are expressed at higher levels in *PVT1-MYC* fusion group 3 MBs compared to non-fusion cases. miR-1204 inhibition reduced proliferation of group 3 MB cells at a level comparable to *MYC* knockdown, while an MB cell line with neither *MYC* amplification nor detectable *PVT1-MYC* fusion gene was unaffected by miR-1204 knockdown (119). *PVT1* stabilizes MYC protein to promote tumorigenesis, and the *PVT1* locus is often amplified in breast cancer (120). However, *PVT1* is also frequently disrupted by recurrent translocations or deletions in many cancer types, including MB, suggesting that it may have additional regulatory functions. Of note, a recent study showed that *PVT1* lncRNA expression was not required to inhibit *MYC* transcription; instead, the *PVT1* promoter competed with *MYC* for enhancer binding at the *PVT1* locus, preventing *MYC* promoter firing and suppressing transcriptional elongation of the *MYC* oncogene and reducing cancer cell growth (121). This might indicate a lncRNA-independent tumor-suppressive role for the *PVT1* promoter in MB and suggest that regulatory sequences in lncRNA genes may contribute to tumorigenesis.

Zhang *et al.* measured *HOTIAR* expression in human clinical MBs samples and cell lines and found that *HOTAIR* expression is higher in MB tissues and cell lines than normal samples. *HOTAIR* can promote MB cell growth, migration, invasion, EMT and inhibit cell apoptosis by negatively regulating miR-1 and miR-206 and activating YY1 expression (122). *CRNDE* lncRNA was found to be highly upregulated in cisplatin-treated MB cells, and it promoted tumor proliferation, apoptosis, migration, invasion and resistance to chemotherapeutics. Mechanistically, *CRNDE* functions as a ceRNA that directly binds to and negatively regulates miR-29c-3p, thereby mediating MB growth and drug resistance (123).

The recent oncogenic lncRNA characterized in MB was *TP73-AS1* (124), a transcript antisense to p73 mRNA encodes for a p53 TF family member. p73 is implicated in brain development (125) and cancer (126), including MB (127). Comparative expression analyses of *TP73-AS1*, using the R2 platform1 and the Cavalli cohort (128), revealed its upregulation in SHH-MB, where TP73-AS1 promoter is significantly hypomethylated (125). In SHH-MB cells, Varon *et al.* tested the role of *TP73-AS1* and found that knocking down *TP73-AS1* inhibited cell proliferation, cell migration and increased apoptosis, irrespective of *TP53* mutant status. Supporting the genetic independence of *TP73-AS1* and P53 family, the TP73 knockdown did not impact the levels of *TP73-AS1*. *In vivo* experiments showed that knocking down *TP73-AS1* reduced tumor growth, leading to longer survival time in tumor-carrying mice (124). Li *et al.*, found *TP73-AS1* acts as a potential oncogene and regulates proliferation, migration, invasion and apoptosis by targeting miR-494–3p in MB cells (129).

Our group has recently analyzed publicly available microarray and RNA-seq datasets by using machine-learning statistical algorithms (random-forest and Lasso) to identify a group of putative lncRNA signatures that may be able to differentiate medulloblastoma subgroups accurately (130). Among those, we found *lnc-HLX-2–7* lncRNA was highly upregulated in group 3 MBs compared to other groups. Knockdown of *lnc-HLX-2–7* significantly reduced cell growth and induced apoptosis *in vitro* and *in vivo*. *lnc-HLX-2–7* regulates modulation of cell viability, cell death and energy metabolism signaling pathways (131). Table 2 summarizes the lncRNAs implicated in MB and their molecular partners or genomic targets that mediate MB phenotypes of proliferation, growth suppression, migration, invasion and metastasis.

**Table 2.** Summary of lncRNAs involved in epigenetic regulation and in medulloblastoma biology

lncRNA	Biological roles in medulloblastoma	Molecular functions	Target pathway	References
*ANRIL*	Promotion of cell proliferation and migration	miRNA sponge (miR-323)	p38 MAPK, ERK, AKT, Wnt pathway	(217)
*CCAT1*	Promotion of cell proliferation and metastasis	Unknown	MAPK pathway	(218)
*CRNDE*	Promotion of cell cycle progression and chemosensitivity	miRNA sponge (miR-29c-3p)	Unknown	(123,219)
*HOTAIR*	Promotion of cell proliferation, migration and invasion	miRNA sponge (miR-1 and miR-206)	Unknown	(122)
*linc-NeD125*	Promotion of cell proliferation, migration and invasion	miRNA sponge (miR-19a-3p, miR-19b-3p and miR-106a-5p)	Unknown	(118)
*LOXL1-AS1*	Promotion of cell proliferation and metastasis	Unknown	PI3K/Akt pathway	(220)
*Nkx2–2as*	Suppression of cell proliferation, migration and invasion	miRNA sponge (miR-103, miR-107 and miR-548m)	Shh pathway	(75)
*SPRY4-IT1*	Promotion of cell proliferation, migration and invasion	Unknown	Shh pathway	(221)
*TP73-AS1*	Promotion of cell proliferation, migration and invasion	miRNA sponge (miR-494–3p)	Unknown	(124,129)
*UCA1*	Promotion of cell proliferation and migration	Unknown	Unknown	(222)

### Epigenetic control of lncRNA expression in brain tumors

Overexpression or repression of key lncRNAs can be mediated by aberrant DNA methylation or histone modifications at their gene promoters. Dysregulated expression of such lncRNAs may further contribute to tumorigenesis through aberrant epigenetic changes in gene regulatory mechanisms. Emerging evidence indicates that lncRNAs constitute a network of epigenetic modulators by forming platforms for chromatin-remodeling complexes and transcription factors capable of regulating the transcriptional state of lncRNA-controlled genomic loci (132,133). Several studies have demonstrated that lncRNA expression can be controlled by DNA methylation at promoter regions and by direct interaction with histone modifiers and chromatin-remodeling complexes (33,134,135). *MEG3* downregulation in gliomas was recently found to be due to hypermethylation of its promoter. Treatment of glioma cells with the DNA methylation inhibitor 5-AZA-2'-deoxycytidine decreased aberrant promoter hypermethylation and prevented the loss of *MEG3* expression. DNMT1 expression is inversely correlated with the *MEG3* transcript, supporting DNMT1’s involvement in *MEG3* dysregulation in gliomas (112). Importantly, the same association between *MEG3* expression and promoter methylation has been observed in hepatocellular carcinoma, and direct involvement of *miR-29a*, a DNMT1 and DNMT3B epigenetic modulator, was also demonstrated (136). The activity of the tumor suppressor *ADAMTS9-AS2* is also controlled by DNA methylation in gliomas, and its expression is positively correlated with ADAMTS9 and DNMT3A expression (137). A recent study showed regulation of *LOC285758* expression by DNA methylation that differed within glioma subtypes, with overexpression and higher promoter hypomethylation in glioblastomas (138).

Histone modifications play an essential role in the regulation of lncRNA expression. LncRNA promoters exhibit specific histone marks, including trimethylated H3K4 (H3K4me3), H3K27 (H3K27me3), H3K36 (H3K36me3), and acetylated H3K9 (H3K9ac) and H3K27 (H3K27ac), suggesting that they undergo epigenetic regulation similar to that of protein‐coding genes (139). Genome-wide analysis in different cell and tissue types has revealed that highly expressed lncRNAs were enriched in active H3K4me3 and H3K36me3 sites, whereas those with low expression were highly marked by H3K27me3. Zhao *et al.* proposed an integrative method to identify epigenetically altered lncRNAs and their associated histone modifications. By combining RNA- and ChIP-seq data for H3K4me3 and H3K27me3 marks, the authors found that many disease-related lncRNAs were associated with aberrant epigenetic modifications and that their neighboring genes were enriched for PRC2-binding sites (140). For example, fetal tissues and glioblastoma expressed high levels of *HOTAIRM1* (141) and showed a gain of H3K4me3 in glioma stem cells compared to in glioblastoma multiforme tissue. Epigenetically activated *HOTAIRM1* can also negatively or positively affect the expression of its neighboring genes. Indeed, *HOTAIRM1* plays key roles in myeloid differentiation by *cis*‐regulating the expression of neighboring *HOXA* genes (142). In this way, H3K4me3‐activated *HOTAIRM1* could contribute to glioblastoma development by enhancing the expression of neighboring *HOXA1*, an oncogene present in several cancers. Hyperacetylation of H3K9 in the promoter region may account for the upregulation of *CRNDE*, a key lncRNA involved in neurogenesis and probably also in gliomagenesis. H3K9ac is a known mark for actively transcribed promoters, and the acetyltransferase P300/CBP protein responsible for its acetylation was shown to be recruited to the *CRNDE* promotor in glioblastoma cells but not in normal glial cells (143).

Epigenetic modulators such as BRD4 (member of BET‐bromodomain and extra terminal domain) proteins with histone acetyltransferase and chromatin remodeling functions upregulate *HOTAIR* expression in glioma cells. ChIP analysis demonstrated that BRD4 binds to the *HOTAIR* promoter, suggesting that BET proteins can directly regulate *HOTAIR* expression. Moreover, the treatment of glioblastoma cells with the BET inhibitor I‐BET151 restored the expression of several other glioblastoma-downregulated lncRNAs. Conversely, *HOTAIR* overexpression in conjunction with I‐BET151 treatment abrogated the BET bromodomain inhibitor's anti-proliferative activity in glioblastoma cells (64). Also, several lncRNAs function as scaffolds for the PRC2 complex and guide these complexes to their endogenous targets. *HOTAIR* regulated cell cycle progression in glioma cells via interactions with EZH2, and inhibition of *HOTAIR* repressed glioblastoma tumor growth *in vivo* (144).

**Figure 2.**
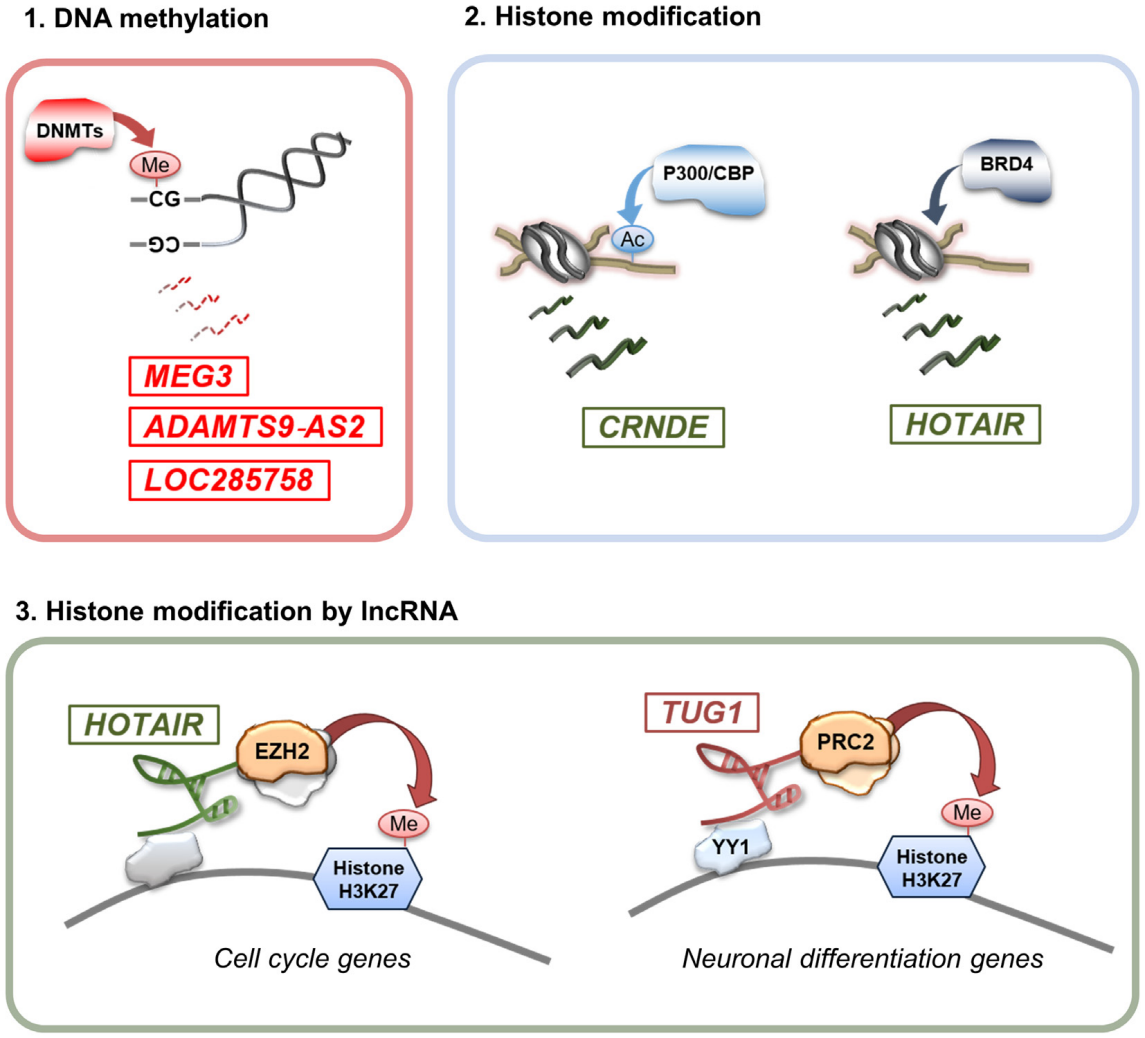
Crosstalk between epigenetic modulation and lncRNAs in brain tumors. (**1**) Epigenetic silencing through DNA methylation at the promoter regions of tumor suppressors *MEG3*, *ADAMTS9‐AS2* and *LOC285758*. (**2**) Histone modifier p300/CBP protein enhances acetylation of H3K9 at the *CRNDE* promoter. BRD4 binds to the *HOTAIR* promoter and directly regulates *HOTAIR* expression. (**3**) *HOTAIR* regulates cell cycle progression in glioma cells via interactions with EZH2. *TUG1* promotes locus‐specific methylation of histone H3K27 via YY1 binding.

*TUG1* represents another example of complex epigenetic gene regulation by lncRNAs. In brain development, by binding to PRC2 components, *TUG1* selectively regulates neuronal differentiation-associated genes. In gliomas, *TUG1* promotes locus-specific methylation of histone H3K27 via YY1 binding (87). The YY1 and PRC2 binding regions of *TUG1* are evolutionarily conserved in mice and humans, suggesting their importance for *TUG1* function in tumorigenesis (145). The large number of epigenetically dysregulated lncRNAs reveals that epigenetic inhibitors may induce anti-tumor effects in brain cancers by modulating lncRNA networks. Thus, the expression of lncRNAs is mediated by DNA methylation and histone modifications, while lncRNAs themselves dynamically regulate epigenetic modifications, both normally and in brain tumors (Figure 2).

### Therapeutic potential of lncRNAs in brain tumors

New findings on lncRNAs in brain tumors are beginning to be incorporated into clinical trial data. LncRNAs may affect clinical research and patient management in several ways. Some data associate the expression of certain lncRNAs with patient prognosis (146). Genome-wide lncRNA expression profiles in glioma patients have revealed that dysregulated lncRNA expression plays important role in tumorigenesis and malignant progression, and several lncRNAs are related to prognosis patients with glioblastoma (147).

For glioma patients, surgery followed by chemotherapy is an effective treatment. However, the efficacy of chemotherapy remains poor due to the development of drug resistance (148). Suppressing drug resistance in glioma cells is thus a challenge for the treatment of glioblastoma (149,150). Accumulating evidence confirms that dysregulated lncRNAs play important roles in the drug resistance of many cancer types, including glioma.

Liu *et al.* reported changes in the expression levels of several lncRNAs in response to genotoxic stress. By detecting the expression patterns of lncRNAs in human glioma cell lines treated with doxorubicin and resveratrol, they found that *GAS5* was upregulated during doxorubicin-induced apoptosis, *NEAT1* and *MIR155HG* were upregulated in response to resveratrol-induced apoptosis, and *MEG3* and *ST7OT1* are upregulated in response to apoptosis induced by both agents. *TUG1*, *BC200* and *MIR155HG* were downregulated during necrosis induced by high-dose doxorubicin. These results may indicate that cellular defenses against genotoxic agents are controlled by distinct lncRNAs (151).

TMZ-based chemotherapy is one of the most widely used treatments for glioma and its use has contributed to overall survival gains in glioblastoma patients (152,153). Several lncRNAs have been found to be involved in chemoresistance to TMZ in glioma cells. *H19* is significantly upregulated in TMZ-resistant glioma, and *H19* knockdown decreased the IC50 values for TMZ and increased rates of apoptosis in TMZ-resistant glioma cells by altering the expression of major drug resistance genes (154). *MALAT1* reduced the sensitivity of glioma cell lines to TMZ by enhancing EMT and upregulating multidrug resistance-associated protein expression (155). *CASC2* sensitized glioma cells to TMZ and amplified the TMZ-induced suppression of cell proliferation by upregulating PTEN protein expression and downregulating phosphorylated AKT through miR-181a (156).

The DNA repair proteins MGMT and SP1 play important roles in TMZ resistance and are upregulated in TMZ-resistant glioma cell lines (157). MiR-29c regulated *SP1* and *MGMT* expression to enhance glioma cell chemosensitivity to TMZ. *XIST* directly targeted miR-29c and inhibited miR-29c expression from amplifying glioma cell line chemoresistance to TMZ (158). These findings indicate that lncRNAs have potential as therapeutic targets in TMZ-resistant glioma. Circulating levels of *HOTAIR* are significantly correlated with high‐grade brain tumors, and one study demonstrated that this lncRNA could be considered a novel prognostic and diagnostic biomarker for glioblastoma (159). Moreover, aberrant lncRNA expression is correlated with response to therapy in glioma *in vitro* and *in vivo*. For example, *MALAT1* knockdown increased the permeability of the blood‐tumor barrier, which might help to improve tumor penetration with therapeutics (160), while restoration of *CASC2* expression upregulated PTEN and increased glioma sensitivity to TMZ‐based chemotherapy (161).

There are also examples of lncRNA in MB, leading us to distinguish subgroups of this tumor more precisely. The two most heterogeneous, and otherwise closely related and difficult to distinguish, MB subgroups are groups 3 and 4. It has been reported that some lncRNAs, such as *ARHGEF7-AS2*, *lnc-HLX-1*, *lnc-EXPH5–2*, *lnc-CH25H-2* and *lnc-TDRP-3*, demonstrate differential expression in these two groups, and that other lncRNAs were subgroup-specific: *lnc-CCL2–2* in the WNT subgroup, *lnc-ABCE1–5* in the SHH subgroup, *USP2-AS1* in group 3 and *lnc-TBC1D16–3* in group 4 (130). Another lncRNA, *lnc-FAM84B-15* (*CCAT1*), was upregulated in WNT and group 3 of MB, activating the MAPK pathway and significantly stimulating proliferation and metastasis (162).

**Figure 3.**
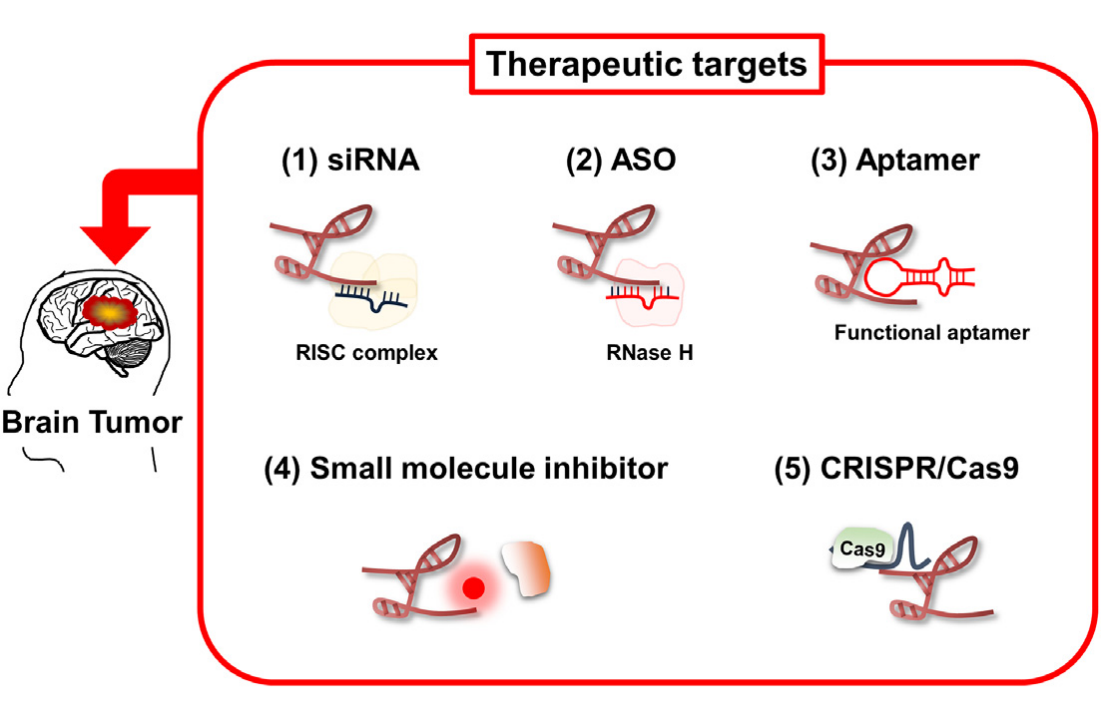
Therapeutic potential of lncRNAs in brain tumors. (**1**) Small interfering RNA (siRNA) are double-stranded RNA oligonucleotides antisense and complementary to target lncRNA sequences. They induce degradation of their target by recruiting the RISC (RNA-induced silencing) complex. (**2**) An antisense oligonucleotide (ASO) is a single-stranded DNA oligonucleotide that is complementary to the target RNA and able to induce its degradation by recruiting RNase H. (**3**) Aptamers are single stranded DNA folded into secondary and tertiary structures that can bind specific structural regions of the target lncRNAs. (**4**) Small molecule inhibitors can disrupt lncRNA interactions. (**5**) Genome editing strategies such as CRISPR/Cas9 are powerful tool to knock-in or knock-out lncRNA candidates.

Compared to protein-coding transcripts, targeting lncRNAs is challenging. Lack of protein products means that only RNA-based tools are usable. Unlike proteins with specific domains that are easy to target with small molecule drugs, lncRNA conformations are poorly understood, making structure-based strategies difficult to develop. However, several approaches have been proposed to reduce the expression of lncRNAs or inhibit their function (Figure 3).

One of the most explored methods to inhibit lncRNAs is the delivery of synthetic oligonucleotide-based molecular products (163). Small interfering RNAs (siRNAs) are double-stranded RNA oligonucleotides, antisense and complementary to target lncRNAs. They induce the degradation of their target by recruiting the RISC (RNA-induced silencing) complex. SiRNA targeting *HOTAIR* has been shown to suppress the progression of endometrial carcinoma *in vivo*, demonstrating that targeting *HOTAIR* can be a novel therapeutic strategy for endometrial cancer (164). The delivery of nanoparticles containing siRNAs against lncRNA *DANCR* prolonged the survival of AML (Acute Myeloid Leukemia) mouse model (165). Antisense oligonucleotide (ASO) are single-stranded DNA oligonucleotides duplexing by base complementarity their target lncRNAs to promote their degradation by RNase H (166,167). For example, a study showed that using ASOs targeting *TUG1* coupled with a drug delivery system induced glioma cell differentiation and repressed tumor growth *in vivo* (87). Aptamers could also provide greater specificity. The chemical structure of aptamers could be modified to enhance their stability and half-life. Aptamer-based -therapeutics are undergoing clinical trials (non-small cell lung cancer, renal cell carcinoma and AML) (168). Fatemi *et al.* proposed innovative therapeutic strategies based on small molecule inhibitors that disrupt lncRNA–protein interactions (169,170). New genome editing strategies such as CRISPR/Cas9 seem to be powerful tools to knock-in or knock-out lncRNA candidates (171). Modified CRISPR systems could also generate cytidine substitution into uridine to correct oncogenic single nucleotide polymorphisms (SNPs), knowing that numerous SNPs have been associated with potential predictive biomarkers for the risk of cancer, including SNPs in *ANRIL*, *MALAT1*, *HULC* and *PRNCR1* lncRNA (172,173).

The ongoing case-control observational study sponsored by Peking Union Medical College Hospital (NCT03738319) will analyze the expression of miRNAs and lncRNAs in blood samples from 160 patients with high grade serous ovarian cancer and benign gynecologic diseases. Differential expression of miRNAs and lncRNAs will be compared between cancer and control groups, and candidate ncRNAs will be validated as biomarkers for the detection and prognosis of ovarian cancer. Hopefully, completing this trial will lead to promising clinical lncRNA biomarkers that can aid in early detection of this disease, which is often not diagnosed until an advanced stage (174).

*H19* is a lncRNA with several oncogenic mechanisms of action and is often upregulated in different types of cancer (175). Due to its upregulation in cancer cells, researchers have used its promoter to deliver a cancer-specific expression of downstream sequences. One compound, BC-819 (also known as DTA-H19), is a DNA plasmid expressing diphtheria toxin A (DTA) under the regulation of the *H19* promoter, delivered as a complex with polyethyleneimine (PEI). BC-819 has completed phase 1/2a trials in pancreatic, ovarian and bladder cancers, with current clinical development focused on bladder cancer, where *H19* is often suppressed in normal cells but upregulated in tumor cells (176). While not directly targeting an lncRNA, this is an interesting example of how ncRNAs properties, like *H19* cancer-specific expression, can be harnessed for novel cancer therapeutic interventions.

## CONCLUSION

Despite major efforts directed toward improving brain tumors treatment, prognosis remains poor and the median overall survival time of patients with high-grade tumors remains abysmal, even with aggressive surgery, radiotherapy and chemotherapy. A lack of available tools for early diagnosis of brain cancers and the development of resistance to chemotherapy limit current treatment efficacy. Recent rapid developments in characterizing the expression profiles and functions of lncRNAs in brain tumors have generated excitement in the field of brain tumor research and therapy over the past few years. Due to the rapid development of high-throughput RNA-seq technology, a set of lncRNAs have been identified as dysregulated in brain tumors and associated with tumor progression and poor clinical outcomes. Therefore, lncRNAs have the potential to serve as biomarkers. The material reviewed showed that lncRNAs could serve as patient biomarkers for tumor types and or grades.

Moreover, lncRNAs play key roles in brain tumor progression, and altering their expression level can affect the proliferation, apoptosis and invasion of glioma cells. A study showed that the lncRNA *TUG1* responded to Notch signaling and promoted the self-renewal of glioma cells and that using ASOs targeting *TUG1* coupled with a drug delivery system induced glioma cell differentiation and repressed tumor growth in vivo (87). This study indicates that lncRNAs have the potential as anti-glioma therapies. As lncRNAs also participate in drug resistance, they could potentially serve as sensitizers when combined with traditional chemotherapy. LncRNA-based cancer therapies are promising. Future work must be needed to screen for candidate lncRNAs and develop delivery strategies to translate these molecular data to the clinic.

A recent study functionalized lncRNAs in drug resistance by integrated genome-wide CRISPR activation of 14 701 lncRNAs and found that growth arrest-specific six antisense (*GAS6-AS2*) triggered hyperactivation of the GAS6/TAM (TYRO3-AXL-MER2K) pathway, which is a known drug resistance mechanism in multiple cancers (177). With further identification of the molecular mechanisms of lncRNAs, especially the regulatory networks between lncRNAs and other oncogenic and tumor suppressor genes, we postulate that soon lncRNAs could be used as novel biomarkers and therapeutic targets for patients with brain tumors.
